# The spectrum of skin diseases in a rural setting in Cameroon (sub-Saharan Africa)

**DOI:** 10.1186/1471-5945-12-7

**Published:** 2012-06-21

**Authors:** Anne-Cécile Zoung-Kanyi Bissek, Earnest Njih Tabah, Emmanuel Kouotou, Victor Sini, Faustin N Yepnjio, Rogers Nditanchou, Roland N Nchufor, Defo Defo, Fidèle Dema, Julius Y Fonsah, Alfred K Njamnshi, Walinjom FT Muna

**Affiliations:** 1 Department of Internal Medicine and Specialties, Faculty of Medicine and Biomedical Sciences, The University of Yaounde I, Yaounde, Cameroon; 2 Consultant dermatologist, Mother & Child Center, Chantal Biya Foundation, Yaoundé, Cameroon; 3 Neurology Department, Central Hospital Yaounde, Yaounde, Cameroon; 4 National Leprosy, Buruli Ulcer, Yaws and Leishmaniasis Control Program, Department of Disease Control, Ministry of Public Health, Yaoundé, Cameroon; 5 Dermatologist, District Hospital, Biyemassi, Yaounde, Cameroon; 6 CHU, Limoges, France; 7 Faculty of Medicine and Biomedical Sciences Yaounde, Yaounde, Cameroon; 8 Sa’a District Hospital, Sa’a, Centre Region, Cameroon; 9 Head of Neurology Dept, Central Hospital Yaounde, PO Box 25625, Yaounde, Cameroon; 10 Head of Department of Internal Medicine and Specialties Faculty of Medicine and Biomedical Sciences, The University of Yaounde I, Yaounde, Cameroon

**Keywords:** Skin diseases, Rural communities, Epidemiology, Sub-Saharan Africa

## Abstract

**Background:**

Skin disorders are generally considered to be more prevalent in the rural areas of Cameroon. This study was carried out to verify this assumption by describing the spectrum of skin disorders in a rural setting of Cameroon.

**Methods:**

We carried out a community-based clinical skin examination of 400 consenting subjects from 4 villages of Cameroon: Nyamanga (27%), Yebekolo (24%), Mbangassina (23%) and Bilomo (26%).

**Results:**

The overall prevalence of skin diseases in our sample was 62% {95% CI: 57.2%, 66.8%} (248/400). The commonest skin disorders were: fungal infections (25.4%), parasitic infestations (21.4%), atrophic skin disorders (11.7%), hypertrophic skin disorders (9.7%), disorders of skin appendages {acne} (8.9%), benign neoplasm (6.5%), bacterial skin infections (5.2%), pigmentation disorders (4.8%), and dermatitis/eczema (4.0%). Skin infections and infestations constituted 52.82% of all skin disorders. The overall prevalence of infectious and parasitic infestation was 32.75% {95%CI: 28.17%, 37.59%} (131/400) as against 29.25% {95%CI: 24.83%, 33.98%} (117/400) for non-infectious disorders.

Among people with skin infections/parasitic infestations, those with fungal infections and onchocercal skin lesions were the most prevalent, accounting for 48.1% (63/131) and 35.1% (46/131); and an overall prevalence of 15.75% {95%CI: 12.3%, 19.7%} (63/400) and 11.5% {95%CI: 8.5%, 15.0%} (46/400) respectively.

There was secondary bacterial infection in 12.1% {95%CI: 8.31%, 16.82%} (30/248) of subjects with skin diseases. Hypertrophic and atrophic disorders of the skin were mainly keloids (9.68%), scarification marks (6.05%) and burn scars (5.65%). Skin diseases like dermatitis and eczema (4.03%), malignant tumours and pigmentation disorders were rare in our sample.

The proportion of subjects diagnosed with skin disorders after examination (62.8%) was significantly higher than the proportion of 40.8% that declared having skin diseases (p < 0.0001).

**Conclusion:**

The prevalence of skin diseases in the rural Mbam valley is alarming, dominated by easily treatable or preventable skin infections and their magnitude is highly neglected by the community, contrasting with findings in the urban setting. Similar studies are needed in other ecological/demographic settings of the country in order to construct a better understanding of the epidemiology of skin disorders. This would lead to the development of national policies to improve skin care.

## Background

Generally, the information available on the prevalence and incidence of common skin diseases is scarce. This is even more so in Sub-Saharan Africa (SSA). However, since the last WHO 30-year review of epidemiological studies in which only 18 were published on common skin diseases in the developing world [1], with 10 of them from SSA, only 7 studies have been carried out in the region.

In Cameroon, a recent hospital-based study estimated the prevalence of common skin disorders among patients consulting the dermatology unit of a reference hospital in the capital city of Yaoundé as follows: allergic reactions (34.3%), infections (19.6%), skin appendages disorders (14.6%) and pigmentation disorders (4.3%) to be the most frequent common skin disorders [2]. Although there are reports of an invasion of allergic diseases in developing countries [3-5] the inherent selection bias in our survey in Yaoundé by virtue of its hospital-based design, does not permit us to generalize the findings to the community. Furthermore, the profile of common skin disorders has been noted to be different in the rural areas, where it is dominated by infections, allergies representing only a small proportion. For example, in a community-based survey of common skin diseases in Tanzania, Gibbs determined an overall prevalence of 26.9%, the bulk (73.9%) of which was related to transmissible disorders, and occurring mainly in children [6]. In Northern India, Dogra and Kumar reported a point prevalence of at least one skin disease of 38.8% among children in community with infections as the most common (11.4%) [7].

Skin disorders have been generally considered to be more prevalent in the rural areas of Cameroon where poor hygienic conditions prevail and access to health care is limited. However, no study has yet been carried out to verify this assertion. We formulated the hypothesis that skin disorders are more frequent in the rural areas of Cameroon than in the urban areas. We therefore carried out this study with the purpose of describing the spectrum of skin disorders in a rural setting of Cameroon.

## Methods

**Figure 1:**
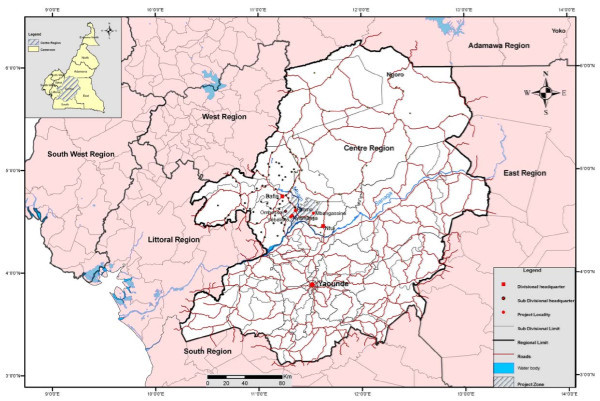
The administrative map of Cameroon, highlighting the Mbam and Kim division (study area) of the Centre Region.

**Figure 2:**
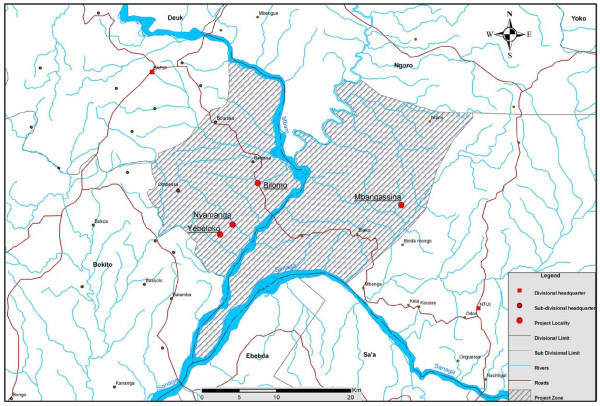
The partial map of Mbam and Kim Division showing the 4 survey villages in the study area (shaded).

In March 2010, we carried out a community-based cross-sectional survey of skin disorders in 4 rural communities (Bilomo, Mbagassina, Nyamanga and Yebekolo) located some 120 Km north of Yaoundé the capital city of Cameroon. These villages are found in the Mbangassina Subdivision of the Mbam and Kim Division in the northern part of the Central Region. The study site is found in the forest savanna zone with a warm and humid tropical climate and several rivers flow through it (see Figures 1 and 2). The people who live in the villages belong to several closely related ethnic groups and practice peasant farming and fishing.

The survey was done within the framework of a health campaign organized conjointly by a multidisciplinary team from the Department of Internal Medicine and Specialties of the Faculty of Medicine and Biomedical Sciences of the University of Yaounde I and the Nyamanga Catholic Health Centre.

The health campaign team visited the 4 communities according to a pre-established schedule that was communicated to the communities through the media of churches and community social group meetings, several times before the appointed day. The team that was constituted of 3 dermatologists, 2 neurologists, 3 general physicians, 2 medical students, 1 psychologist, and 2 nurses, proceeded with free clinical consultations (for skin conditions and epilepsy) and examination of villagers who came to the community health post in each community.

The dermatology team (one senior and two juniors) was assisted by the two medical students for the clinical examination. Each disease registered was classified on the basis of the WHO International Classification of Primary Care (ICPC) [8]. In order to bring out the primary skin disorders in the study sample, in cases of secondary bacterial infection, only the primary dermatosis was taken into consideration for data analysis. Nevertheless, they were documented and treated separately. The diagnosis of the medical conditions was based essentially on clinical observations, as no laboratory investigation was undertaken. Information on skin diseases was recorded using a questionnaire designed for the survey. Consent was obtained from study subjects for publication of photographs of skin lesions.

Data was entered using Epi Info version 3.5.1 for Windows and analyzed using SPSS version 15.0. Descriptive statistics including frequency, means, and proportions were used; and the 95% confidence interval was calculated for proportions. The Chi-squared test was used to compare proportions and association between categorical variables. The level of significance was set at a P-value of ≤ 0.05. Results were compared with published data from Yaoundé, the capital city of Cameroon.

Ethical clearance (N° 037/CNE/SE/2010) for the survey was obtained from the National Ethics Committee of Cameroon.

## Results

### Socio-demographic characteristics of study subjects

A total of 512 subjects were invited to take part in the survey and 400 (78.13%) gave their consent. The distribution of the subjects according to the villages was as follows: Nyamanga (27%), Yebekolo (24%), Mbangassina (23%) and Bilomo (26%). The male to female ratio was 1:1. Their ages ranged from 9 to 51 years with a mean age of 21 ± 8.13 years, and there was no age difference between males and females (p = 0.269). The largest proportion of the subjects were in the 20–24 years age group (27.75%) followed by the 15–19 years age group (26.0%). They were mostly farmers (47.25%) or pupils/students (37.5%). Although only 17.25% of the participants were married, 35% had at least 1 child. The majority of the subjects (97.25%) had completed at least the primary level of education, with 33.75% having attained secondary education. Most subjects (99%) were Christians.

### Distribution of skin diseases

**Figure 3:**
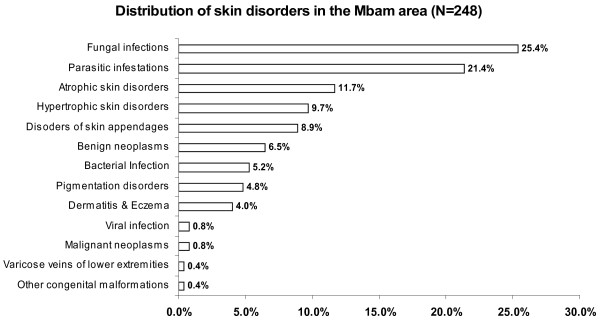
Distribution of skin disorders in the Mbam area.

The details of the skin disorders in our sample are given in Table 1. The overall prevalence of skin diseases in our sample was 62% {95% CI: 57.2%, 66.8%} (248/400). The commonest skin disorders were found to be: fungal infections (25.4%), parasitic infestations 21.4%, atrophic skin disorders (11.7%), hypertrophic skin disorders (9.7%), and disorders of skin appendages {precisely acne} (8.9%), benign neoplasm (6.5%), bacterial skin infections (5.2%), pigmentation disorders (4.8%), and dermatitis/eczema (4.0%) in that order (see Table 1 and Figure 3). Skin infections and infestations constituted 52.82% of all skin disorders, as against 47.18% for non-infectious disorders. The overall prevalence of infectious and parasitic infestation skin disorders was 32.75% {95%CI: 28.17%, 37.59%} (131/400) as against 29.25% {95%CI: 24.83%, 33.98%} (117/400) for non-infectious disorders.

**Table 1:** Distribution of skin disorders by pathological groups

**Pathological group**	**Skin disorders**	**International classification of primary care**	**N**	**Percentage (%)**
**All skin disorders**	**Total**		248	100.00%
** *Fungal infection* **	Dermatophytosis	B 35.9	12	4.83%
	Pityriasis versicolor	B 36.0	51	20.57%
** *Parasitic infestation* **	Onchocerciasis (skin lesions)	B73	46	18.55%
	Scabies	B 86	7	2.82%
** *Atrophic disorders of the skin* **	Scar condition and fibrosis of skin (ethnic scar)	L 90.5	15	6.05%
	Scar condition and fibrosis of skin (Burn scars)	L 90.5	14	5.65%
** *Hypertrophic disorders of the skin* **	Keloid scar	L 90	24	9.68%
** *Disorders of skin appendages* **	Acne	L 70.9	22	8.87%
** *Benign neoplasm* **	Melanocytic Naevi, unspecified	D22.9	16	6.45%
** *Bacterial Infection* **	Superficial impetigo	L01.0	13	5.24%
** *Other disorders of pigmentation* **	Café au lait spots	L 81.3	10	4.03%
	Vitiligo	L 80	2	0.81%
** *Dermatitis and eczema* **	Other atopic dermatitis	L 20.8	6	2.42%
	Seborrhoeic dermatitis	L21	2	0.81%
	Pityriasis rosea	L 42	1	0.40%
	Dry skin	L 85.3	1	0.40%
** *Viral infection* **	Warts	B 07	2	0.81%
** *Malignant Neoplasms* **	Kaposi sarcoma	C 46.9	1	0.40%
	Basal cell carcinoma	C 44	1	0.40%
** *Other congenital malformations of the skin* **	Ichiose vulgaris	Q80.0	1	0.40%
** *Varicose veins of lowers extremities* **	Varicose veins of lowers extremities with ulcer	I 83.0	1	0.40%

**Figure 4:**
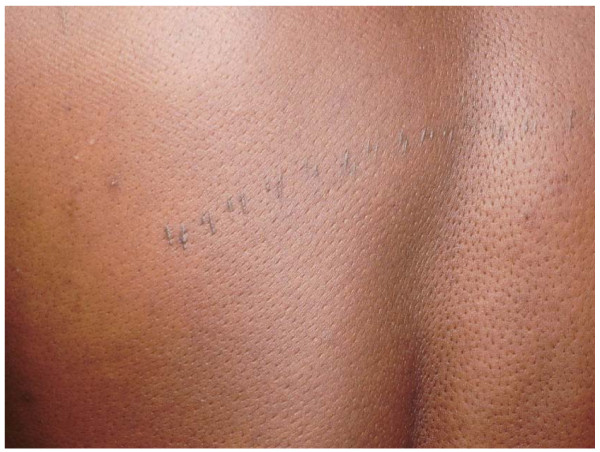
Scarifications.

**Figure 5:**
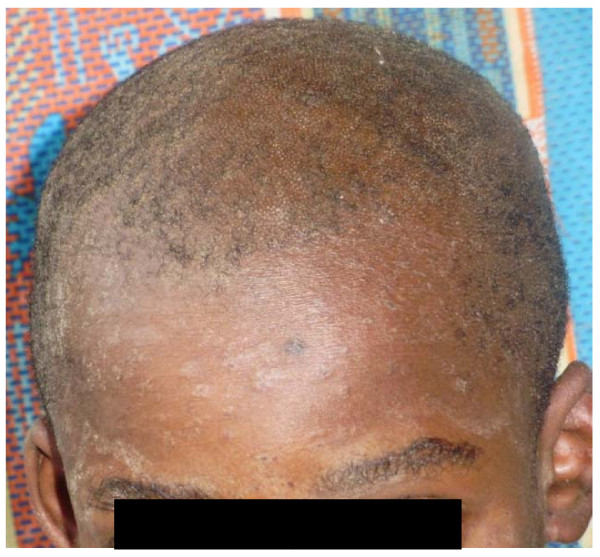
Tinea capitis with involvement of glabrous skin of the face.

**Figure 6:**
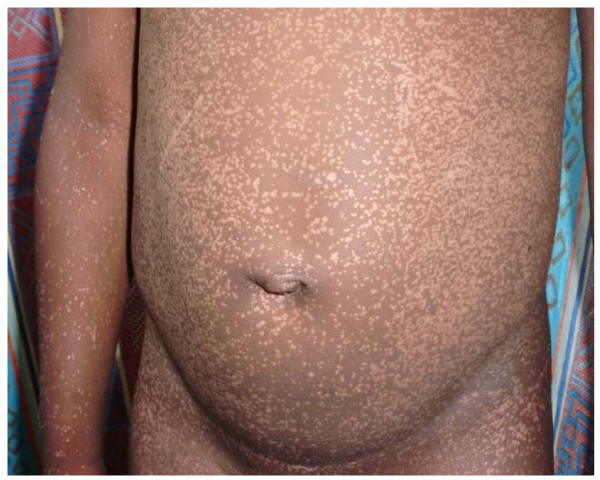
Flat Warts.

**Figure 7:**
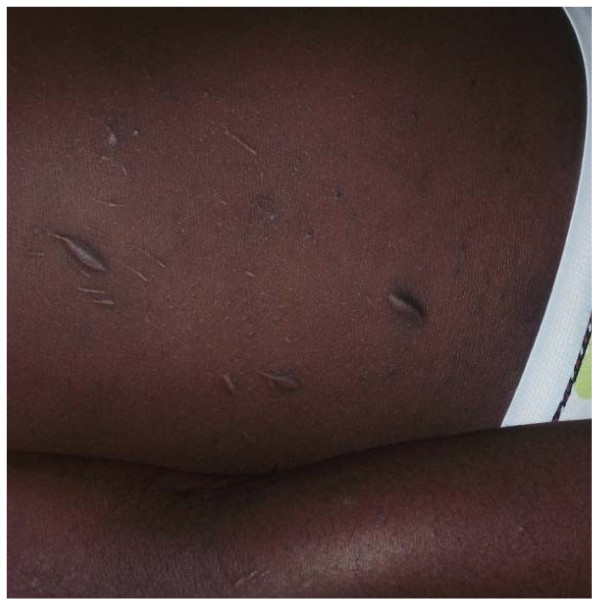
Hypertrophic scars/keloids.

**Figure 8:**
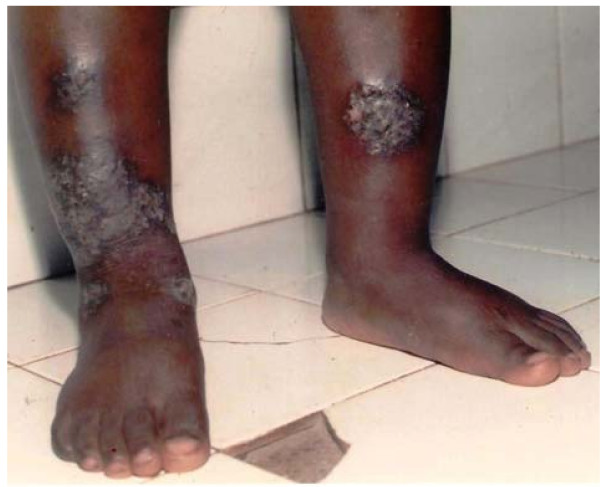
Nummular eczema.

**Figure 9:**
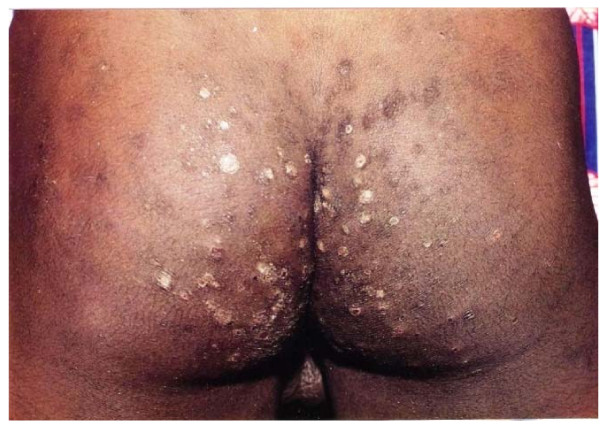
Scabies.

**Figure 10:**
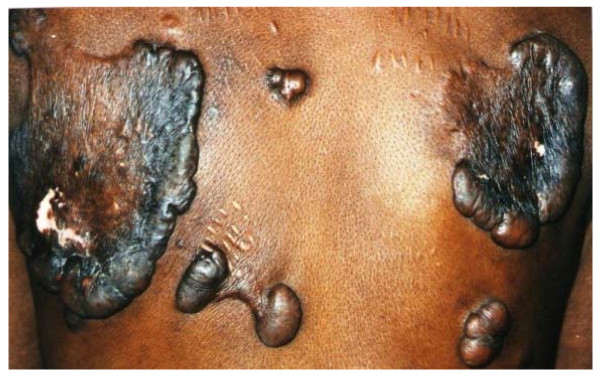
Keloids.

**Figure 11:**
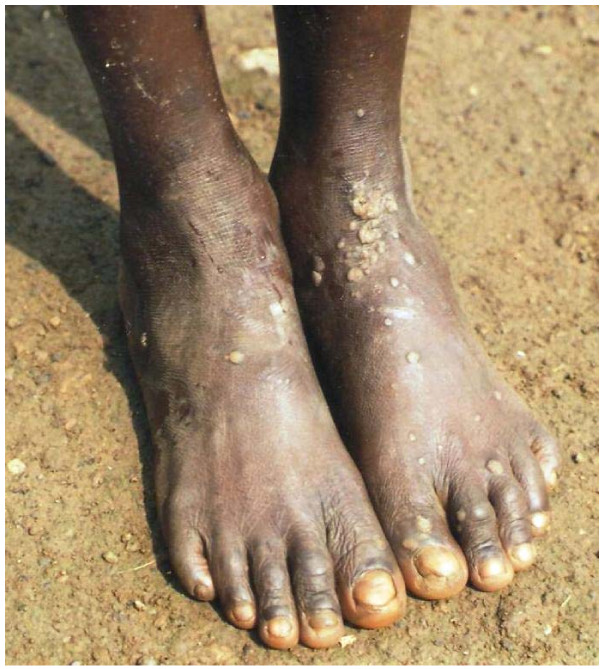
Common warts.

**Figure 12:**
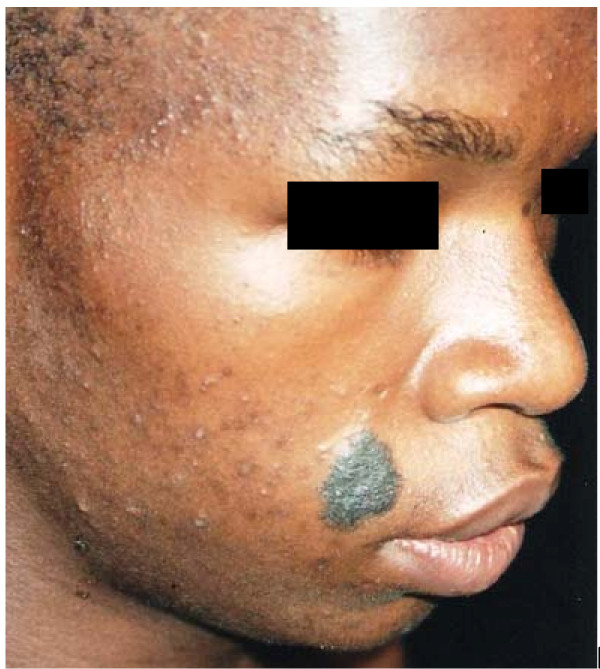
Congenital nevi and acne.

The skin diseases were slightly more prevalent in males 64.0% {95%CI: 56.93%, 70.65%} (128/200) compared to females 60.0% {95%CI: 52.85%, 66.85%} (120/200) although this difference was not significant (P = 0.4099). Among people with skin infections/parasitic infestations, those with fungal infections and onchocercal skin lesions were the most prevalent, accounting for 48.1% (63/131) and 35.1% (46/131); and an overall prevalence of 15.75% {95%CI: 12.3%, 19.7%} (63/400) and 11.5% {95%CI: 8.5%, 15.0%} (46/400) respectively. There was secondary bacterial infection in 12.1% {95%CI: 8.31%, 16.82%} (30/248) of subjects with skin diseases (result not shown as this group did not constitute part of the data analysis). Hypertrophic and atrophic disorders of the skin were mainly keloids (9.68%), scarification marks (6.05%) and burn scars (5.65%). Skin diseases like dermatitis and eczema (4.03%), malignant tumours and pigmentation disorders were rare in our sample. Figures 4, 5, 6, 7, 8, 9, 10, 11, 12 show sample pictures of the respective skin disorders recorded in our study population.

The distribution of the top 9 skin disease groups in our sample by demographic variables (Table 2) showed that fungal skin infections were statistically more common in the 10–14 age group (p = 0.032); disorders of skin appendages (acne) were significantly more prevalent in the 20–24 years age group (p = 0.005) while parasitic skin infestations were most common in the above 40 years age group (p = 0.009). Atrophic skin disorders were significantly more prevalent in the females than in males (p = 0.018). Bacterial skin infections were significantly more common in subjects without any education (p = 0.029) while fungal infections were most prevalent in pupils and students (p = 0.003).

**Table 2:** Distribution of top 9 skin disorders (pathological groups) by demographic variables

**Variable**	**Category**	**N**	**Fungal infection**		**Parasitic infestation**		**Atrophic skin disorders**		**Hypertrophic skin disorders**		**Disorders of skin appendages**		**Benign neoplasm**		**Bacterial infection**		**Pigmentation disorders**		**Dermatitis and Eczema**	
			**%**	**P value**	**%**	**P value**	**%**	**P value**	**%**	**P value**	**%**	**P value**	**%**	**P value**	**%**	**P value**	**%**	**P value**	**%**	**P value**
*Total*		248	25.4		21.4		11.7		9.7		8.9		6.5		5.2		4.8		4.0	
*Age*	5 – 9 yrs	6	33.3		16.7		16.7	0.097	0.0	0.529	0.0		16.7	0.262	16.7	0.543	0.0	0.738	0.0	
	10 – 14 yrs	42	**38.1**	**0.032**	11.9		14.3		14.3		2.4		4.8		2.4		4.8		2.4	
	15 – 19 yrs	60	36.7		18.3		10.0		10.0		6.7		3.3		8.3		3.3		1.7	
	20 – 24 yrs	70	18.7		18.6		4.3		12.9		**21.4**	**0.005**	8.6		5.7		4.3		2.9	
	25 – 29 yrs	40	12.5		32.5		22.5		2.5		5.0		7.5		2.5		10.0		5.0	
	30 – 34 yrs	11	27.3		9.1		18.2		9.1		0.0		0.0		9.1		9.1		18.2	0.061
	35 – 39 yrs	8	12.5		25.0		25.0		12.5		0.0		25.0		0.0		0.0		0.0	
	40 yrs +	11	9.1		**63.6**	**0.009**	0.0		0.0				0.0		0.0		0.0		18.2	
*Sex*	Female	120	21.7	0.191	18.3	0.259	**16.7**	**0.018**	11.7	0.305	8.3	0.773	6.7	0.894	5.8	0.686	5.0	0.909	3.3	0.588
	Male	128	28.9		24.2		7.0		7.8		9.4		6.3		4.7		4.7		4.7	
*Marital Status*	Divorced/ Widowed	4	25.0	0.964	25.0	0.720	0.0	0.639	25.0	0.575	0.0	0.333	0.0	0.735	0.0	0.713	0.0	0.867	**25.0**	**0.026**
	Married	34	23.5		26.5		8.8		8.8		2.9		8.8		2.9		5.9		8.8	
	Single	210	25.7		20.5		12.4		9.5		10.0		6.2		5.7		4.8		2.9	
*Religion*	Christianity	244	25.4	0.985	21.7	0.293	11.5	0.404	9.0		9.0	0.529	6.6	0.596	5.3	0.635	4.9	0.649	4.1	0.679
	Islam	4	25.0		0.0		24.0		**50.0**	**0.006**	0.0		0.0		0.0		0.0		0.0	
*Offspring*	0	172	28.5	0.234	19.2	0.215	11.6	0.311	8.7		9.9	0.698	6.4	0.54	5.8	0.635	5.2	0.752	2.3	
	1 – 3	62	17.7		29.0		14.5		6.5		6.5		8.1		4.8		3.2		6.5	
	4+	14	21.4		14.3		0.0		**35.7**	**0.003**	7.1		0.0		0.0		7.1		**14.3**	**0.049**
*Level of education*	None	8	12.5		12.5	0.823	0.0		25.0	0.086	0.0	0.654	12.5	0.501	**25.0**	**0.029**	0.0	0.495	12.5	0.284
	Primary	170	21.2		21.8		**15.3**	**0.032**	11.2		9.4		5.3		5.3		4.1		2.9	
	Secondary	70	**37.1**	**0.025**	21.4		4.3		4.3		8.6		8.6		2.9		7.1		5.7	
*Occupation*	Farmer	116	18.1		24.1	0.278	11.2	0.963	13.8		10.3	0.861	4.3	0.633	7.8	0.312	4.3	0.776	4.3	0.766
	None	35	20.0		17.1		14.3		**14.3**	**0.045**	8.6		8.6		5.7		2.9		2.9	
	Other	10	10.0		40.0		10.0		0.0		10.0		10.0		0.0		10.0		10.0	
	Pupil/Student	87	**39.1**	**0.003**	17.2		11.5		3.4		6.9		8.0		2.3		5.7		3.4	

The proportion of our subjects diagnosed with skin disorders after physical examination (62.8%) was significantly higher than the proportion of 40.8% that declared having skin diseases (p < 0.0001).

## Discussion

The present study presents for the first time in Cameroon, to the best of our knowledge, the spectrum of skin diseases in a rural community. The point prevalence of any skin disease of 62% found in the Mbam area is alarming, and validates the hypothesis of a high prevalence of common skin disorders in the rural community of Cameroon. This prevalence lies in the upper half of the range of 21-87% described in SSA [1]. The prevalence found in our study is comparable to the 64% reported in a community survey in Timor-Leste [9] and 53% reported among school children in Dar es Salaam [10]. However, this prevalence is less than the finding in a similar population in Ethiopia (80.4%) [11]. Nevertheless, it is within two-folds higher than the 26.9% reported by Gibbs in a community-based study of two villages in Tanzania [6] and 38.8% reported by Dogra and collaborators in an Indian community [7]. These data suggest that although the prevalence of common skin diseases is generally high, it varies quite substantially from one geographical area to another and between different segments of the same population. Given that the Mbam area is situated in the equatorial dense forest region of Cameroon with a warm and humid climate, it may not be appropriate to generalize the findings of this study to the whole of Cameroon that has 4 distinct ecological zones. The replication of the study in the other ecological zones of the country is necessary in order to construct a better understanding of epidemiology of skin disorders that will lead to improved care for these conditions in our communities.

In the review by WHO children were found to have more skin diseases compared to adults [1], a finding that was confirmed by the high prevalence rates described among school children in Ethiopia [11], Tanzania [6], Iraq [12], and in the community in Timor-Leste where scabies and pyoderma were more prevalent in children under 10 years of age compared to adults [9]. An analysis of the prevalence rates of common skin disease across different age groups in our sample (Table 2), showed that fungal skin infections were statistically more common in the 10–14 age group (p = 0.032), while disorders of skin appendages (acne) was significantly more prevalent in the 20–24 years age group (p = 0.005) while parasitic skin infestations were most common in the above-40-years age group (p = 0.009). Fungal infections are common in the young as described by Andrews [13]. The prevalence of acne in the 20–24 age group corresponds to acne in adults resulting from bad cosmetologic habits as described by Bissek et al. [14]. Finally, the predominance of parasitic skin diseases in the above-40-years age group is probably related to chronic forms of onchodermatitis in this area with a high endemicity for onchocerciasis [15]. Future studies in a door-to-door strategy may highlight the high prevalence of common skin diseases in this group in Cameroon.

In our study, skin infections and infestations were the commonest skin disorders accounting for 52.82%, followed by scars (21.38%), acne (8.87%), other disorders of pigmentation (4.84%) and dermatitis/eczema (4.03%). Although with varying proportions, the spectrum of skin diseases in our study was similar to that described in other community studies in the rural milieu in which skin infections constituted the bulk [6,7,9].

Superficial fungal dermatitis (25.40%) was the most common skin infection. The predominance of fungal diseases in the category of infections/infestations as shown in Table 1 is not an isolated phenomenon given that many hospital and community-based studies have described it [3,13,16].

Onchocercal skin lesions (18.55%) came second among common skin infections/infestations in our sample. This is unusual as bacterial infections or scabies depending on the context are generally the second most common skin infection described in the literature [3,4]. The Mbam area where our study was based is a well known endemic zone for onchocerciasis [15] and this probably explains this peculiar finding. Secondary bacterial infection was present in about 5.24% of subjects with another skin disease. The repeated scratching in pruritic dermatoses such as fungal and onchocercal skin lesions as well as scabies, usually leads to skin abrasion thus facilitating secondary bacterial infection [1].

Our study demonstrates that common skin diseases constitute a serious cause of morbidity in the Mbam area. This may suggest that the rest of rural Cameroon probably faces the same challenges. This high magnitude of skin disease is grossly under-estimated by the community as only 40.8% of our subjects declared having any common skin disorder compared to the actual finding of 62.8% (p < 0.0001). This situation may be the result of lack of information in the communities on symptoms and eventual complications of common skin diseases. The implication is that some effort still has to be made to improve the level of information in the communities, by organizing sessions of Communication for Behavior Change led by health personnel through the fixed or mobile strategies. These interventions are expected to be effective given that the spectrum of skin disorders found in our study constitutes easily treatable and preventable conditions for the most part.

The prevalence of allergic dermatoses which is far below that of infections/infestations in the Mbam area (4.03% versus 52.82%) as in Bamako, Mali (15.5% versus 58.6%) [17], is strikingly different from the spectrum reported in the Yaoundé hospital-based survey, where allergic skin reactions were the leading cause of skin disorders [2]. Hospital-based surveys in the dermatology unit, suffer from selection bias which according to Mahe [17] and Imudi [18] may be due to the orientation of patients with undiagnosed or chronic dermatoses to specialised centres. Nevertheless, the findings in the Yaoundé reference hospital [14] suggest that the profile of skin disorders in the urban centres is different from that in the rural areas, with a predominance of infections as opposed to allergic skin reactions in the cities. Nnoruka [3] and Ogunbiyi [4] observed a marked change during a comparative study of the spectrum of dermatoses in the hospital-setting in Enugu and Ibadan respectively during a period of 10 years, with a clear predominance of dermatitis especially eczema. The hypothesis proposed for this change has been that of the industrialization of our cities and the constant exposure of the population to allergens [3,19]. Longitudinal population studies will allow a better understanding of this observation.

Although we know that certain skin conditions are closely associated with HIV infection, this was not the focus of our community-based study. Mbuagbaw and collaborators in a descriptive study reported in an urban setting in Cameroon [20] that mucocutaneous infections were the most common problems in a hospital sample of 384 HIV infected subjects. However, the estimated prevalence of HIV infection in the general population [21] (15–49 years) in Cameroon is 5.1% and had been shown to be much lower in the rural areas (6.7% versus 4.0%) [22].

## Conclusion

The prevalence of skin diseases in the rural Mbam valley is alarming, dominated by easily treatable or preventable skin infections, contrasting with findings in the urban setting. The magnitude of skin diseases is highly underestimated or neglected by this community. Similar studies are needed in other ecological and demographic settings of the country in order to construct a better understanding of the epidemiology of skin disorders that will lead to the development of national policies to improve skin care.

## Competing interests

The author(s) declare that they have no competing interests.

## Authors’ contributions

ACZB and AKN conceived the study; AKN, ACZB, ENT, and WFTM designed the study. JYF, RN, RNN, VS, EK, DD, FD and ACZB collected the data. ENT, ACZB, and AKN analyzed the data and wrote the article. FNY and all authors made critical contributions to improve the scientific content of and approved the final draft of the article.

## Pre-publication history

The pre-publication history for this paper can be accessed here:

http://www.biomedcentral.com/1471-5945/12/7/prepub
